# Corrigendum

**DOI:** 10.1002/ehf2.13316

**Published:** 2021-03-17

In the paper by Savarese et al.,^1^ the affiliation of the fifth author, Giuseppe M.C. Rosano was incorrect.

The correct affiliation is shown below.

“Centre for Clinical and Basic Research, Department of Medical Sciences, IRCCS San Raffaele Pisana, Rome, Italy”
